# Apoptosis versus necrosis in tubal ectopic pregnancies following Methotrexate

**DOI:** 10.1111/iep.12465

**Published:** 2023-01-24

**Authors:** Yaron Gil, Asia Zubkov, Jacques Balayla, Aviad Cohen, Ishai Levin

**Affiliations:** ^1^ Department of Gynecology Lis Maternity Hospital, Tel Aviv Sourasky Medical Center Tel Aviv Israel; ^2^ Department of Obstetrics and Gynecology McGill University Montreal Québec Canada; ^3^ The Pathology Institute Tel Aviv Sourasky Medical Center, Sackler School of Medicine Tel Aviv Israel

**Keywords:** apoptosis, ectopic, Methotrexate, necrosis, pregnancy

## Abstract

Methotrexate administration for the treatment of tubal ectopic pregnancies has been shown to cause tubal mass enlargement. Our hypothesis was that, by administrating Methotrexate, a local necrotic reaction occurs, leading to hematoma formation and eventually fallopian tube rupture. Salpingectomy specimens were collected, analysed and divided into three equal groups: patients who received Methotrexate but who ultimately failed medical treatment, patients who had a viable ectopic pregnancy and patients with a self‐resolving ectopic pregnancy that were operated due to other medical indications. The specimens were dyed using the Cleaved Caspase‐3 (Asp175) Rabbit mA. Specimens were divided into three equal groups and analysed. The patients in self‐resolving ectopic pregnancy group were older and had more pregnancies. Rates of apoptosis were found to be less than 1% per slide. Necrosis was not evident in any of the pathological specimens. It seems Methotrexate administration does not lead to a significant tubal necrotic reaction. Further studies are required.

## 1 INTRODUCTION

Systemic, intramuscular (I.M) Methotrexate (MTX) therapy is the treatment of choice for non‐ruptured, haemodynamically stable, ectopic pregnancies. Reported success rates for a single dose MTX therapy are high and are inversely proportional to β‐hCG levels.^1^, ^2^, ^3^ The mechanism of action of MTX is through inhibition of the metabolism of folic acid. By inhibiting the enzyme Dihydrofolate reductase, it prevents purine and thymidylic acid synthesis, which in turn interferes with DNA synthesis, repair and cellular replication. It is an antimetabolite which disrupts actively growing cells, and as such it is used for the treatment of cancer, autoimmune diseases, ectopic pregnancy, as well as other conditions in which rapid cellular proliferation underlies its pathophysiology.^3^ Methotrexate has not only been shown to cause apoptosis in various tissues, such as platelets, but also has been shown to cause necrosis in other tissues, such as renal cells.^4^, ^5^, ^6^ In haemodynamically stable patients with tubal ectopic pregnancies, expectant management and serial β‐hCG follow‐up leading to spontaneous resolution have been repeatedly shown to be a safe and effective treatment alternative, and in fact, in our institution, this so called “watchful waiting,” is the preferred approach for most patients.^7^, ^8^

Previous studies have shown that after MTX administration, there is enlargement of the tubal mass with hematoma formation. This enlargement was shown to persist even up to 63 days and follow the biochemical (β‐hCG) resolution.^9^, ^10^ This hematoma may eventually render the mass unstable and subsequently lead to rupture of the fallopian tube.

Our hypothesis was that when an extra‐uterine pregnancy spontaneously resolves, an apoptotic reaction takes place as part of a physiological process, which leads to a gradual disappearance of the gestational tissue. During this process, hematoma formation and tubal enlargement are limited or do not happen at all, subsequently leading to reduced risk for fallopian tube rupture.

Studies on apoptosis in gestational tissue are sparse. Kokawa et al.^11^ investigated possible effects of implantation on apoptosis. After examining the cleavage of DNA in human chorionic villi and decidua in both intrauterine and ectopic pregnancies, a ladder pattern, which is a histological hallmark of apoptosis, representing breakdown of DNA, was present in the villi of tubal pregnancies. Likewise, an in‐situ analysis revealed that apoptotic cells were predominant in the syncytiotrophoblast in tubal pregnancy. This study demonstrated that apoptosis occurs in the villi, but not in the decidua of tubal pregnancies, unlike the pattern observed in a normal, intrauterine pregnancy. This finding encouraged us to further look for an apoptotic reaction in the fallopian tubes of ectopic pregnancies, and to determine whether differences in apoptotic rates existed on specimens exposed to MTX therapy.

## 2 MATERIALS AND METHODS

All cases of tubal ectopic pregnancies treated in our university affiliated tertiary medical centre between January 2011 and June 2013 were reviewed. The institutional review board “Helsinki Committee” approved the study design, protocol and waiver of informed consent.

A total of 30 pathological specimens of fallopian tubes were randomly selected by serial order and collected. All specimens were embedded in a formaldehyde solution. We divided the patients into three equal study groups: Group “A,” *n* = 10, included haemodynamically stable patients who underwent salpingectomy after treatment failure with MTX. Group “B,” *n* = 10, included haemodynamically stable patients who were operated because of a viable extra uterine pregnancy with a foetal heart rate. Finally, Group “C,” *n* = 10, included patients who had a self‐resolving ectopic pregnancy but were operated according to physician's decision based on relative indications for surgery (ex. patient with a known tubal factor) or due to patients' request.

Treatment failure was defined as constant increase of β‐hCG levels after second dose of I.M MTX. A single pathologist, aware only of the group's name, but blinded to the patient's other personal details, group's classification or the different treatment approaches, dyed and examined all of the specimens and documented the results according to consecutive serial numbers. The specimens were put on slides and dyed using the Cleaved Caspase‐3 (Asp175) Rabbit monoclonal antibody (Cleaved Caspase‐3 antibody, #9579; Cell Signaling Technology). Caspase‐3 is a critical executioner of apoptosis, as it is either partially or totally responsible for the proteolytic cleavage of many key proteins, such as the nuclear enzyme polymerase.^12^ Activation of caspase‐3 requires proteolytic processing of its inactive zymogen into activated p17 and p12 fragments.^13^ The Cleaved Caspase‐3 Rabbit monoclonal antibody recognizes endogenous levels of caspase‐3 protein when cleaved at Asp175. The antibody is produced by immunizing animals with a synthetic peptide corresponding to residues surrounding Asp175 of human Caspase‐3 protein.^14^

The slides were viewed under a light microscope, with an ×100 magnification. The percentage of apoptosis was measured according to the manufacturer's instructions (Cell Signaling Technology). In addition, signs of a necrotic reaction such as generalized swelling of cell membranes, chromatin condensation, breakdown of plasma membranes, infiltration with inflammatory cells and nucleus shrinkage were assessed.

The pathologist performed quantification and registration of the slides. The results were then transferred to the head researcher who uncovered the batch number and separated the results according to the different groups. Data analysis was carried out with analysis of variance for continuous variables and chi‐square for proportions and categorical variables. By convention, statistical significance was defined with a *p*‐value <.05.

## 3 RESULTS

A total of 30 pathological specimens were analysed. After initial assessment, none of the patients was excluded from the study. The patients in group “C,” with the self‐resolving ectopic pregnancy, were older (33.4 vs. 30.8 and 32.3, *p* > .05), had more pregnancies (3.8 vs. 2.4 and 2.4, *p* > .05) and greater history of live born children (Table 1). In addition, they had the lowest β‐hCG levels (Tables 2 and 3) and spent more days in the hospital. Patients in group “C” were also more likely to have conceived spontaneously.

**TABLE 1:** Demographics.

Group	A	B	C	*p*‐Value
Number of patients	10	10	10	>.05
Median age	30.8	32.3	33.4	>.05
Gravidity	2.4	2.4	3.8	>.05
Parity	1.0	0.33	2.1	>.05
Mean gestational week	5.93	6.5	6.61	>.05
Hospitalization days	3.3	2.66	4.6	>.05

*Note*: A—Methotrexate Failure; B—Ectopic Pregnancy with Pulse; C—Self‐resolving Ectopic Pregnancy.

**TABLE 2:** Conception.

Group	A	B	C	*p*‐Value
Spontaneous conception	*n* = 4	*n* = 5	*n* = 7	>.05
Post IVF treatment	*n* = 3	*n* = 3	*n* = 2	>.05
Post IUI/OI treatment	*n* = 3	*n* = 2	*n* = 1	>.05

*Note*: A—Methotrexate Failure; B—Ectopic Pregnancy with Pulse; C—Self‐resolving Ectopic Pregnancy.

Abbreviations: IUI, Intrauterine Insemination; IVF, In Vitro Fertilization; OI, Ovulation Induction.

**TABLE 3:** β‐HCG levels.

Group/Patient number	A	B	C
I	10,660	1088	2537
II	3327	n/a	252
III	2624	18,439	n/a
IV	9422	47,278	2049
V	2607	3905	2195
VI	n/a	n/a	4757
VII	n/a	5400	1700
VIII	7784	1257	1176
IX	1446	n/a	1207
X	2179	n/a	1073

*Note*: A—Methotrexate Failure; B—Ectopic Pregnancy with Pulse; C—Self‐resolving Ectopic Pregnancy.

**FIGURE 1:**
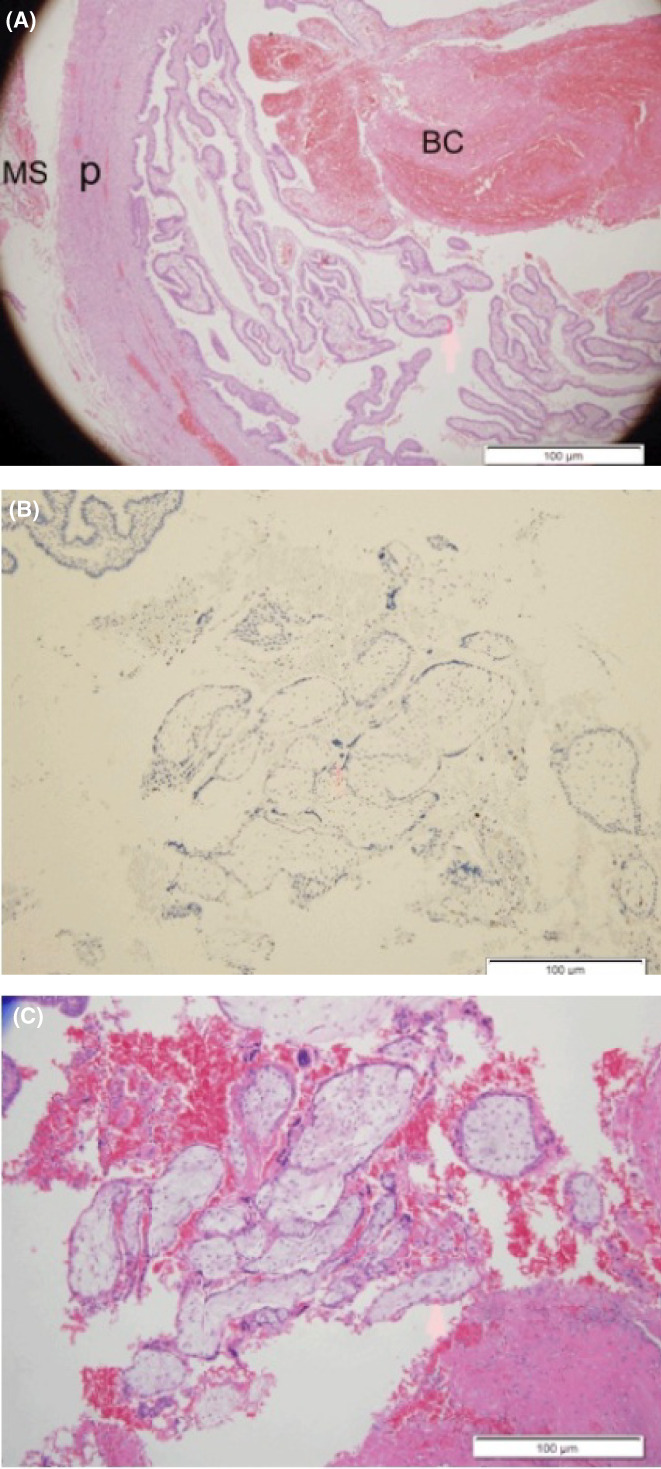
(A) Fallopian Tube, H&E Staining. BC, Blood Clot; MS, Muscular Wall; PL, Plica. Magnification 100×. (B) Villi in Fallopian Tube. The Villi is surrounded by RBC. The outer layer is composed of Syncytiotrophoblast. The inner layer is composed of Cytotrophoblast. The stroma of the Villi contains Hofbauer cells (Macrophages). Haematoxylin and Eosin Stain, Magnification 100×. (C) Villi Fallopian Tube, Same area as Figure 2, Cleaved Caspase – 3 Staining. Red Arrow – Apoptotic Epithelial Cell, Magnification 100×.

After applying the antibody and employing the appropriate smears, all slides were viewed under light microscope. A total of 0–2 apoptotic cells per slide were viewed for all specimens from all study groups, regardless of treatment group. The subsequent apoptotic rate was lower than 1% (Figure 1A–C). Histological signs of necrosis were not evident in either of the pathological specimens. Due to the low number of cells identified, these findings did not confer any statistical significance. It was observed that apoptosis can be found in trophoblastic cells of tubal ectopic pregnancies after salpingectomy which is not the regular component found in trophoblast turnover.^15^

## 4 DISCUSSION

To the best of our knowledge, this was the first attempt to examine the cellular reaction in the fallopian tubes after systemic MTX therapy for tubal ectopic pregnancies. Our objective was to demonstrate that MTX, which is a common, widely used treatment for patients with tubal ectopic pregnancies may actually lead to a pathological process in the fallopian tube, thus rendering it more friable, subsequently increasing the chances of fallopian tube rupture. That is in contrast to a more physiological process, possibly apoptosis, of a self‐ resolving ectopic pregnancy. Elkholy et al.^16^ have examined the effect of intraperitoneal MTX in rats on fallopian tubes and found that in high doses it can induce long‐term irreversible damage. In contrast, Kooi et al.^17^ showed that even injecting MTX directly into the fallopian tube does not cause damage or make it more susceptible to rupture, though this study included only five specimens and so it is unclear if the sample is sufficient to reach a valid conclusion.

Unfortunately, the results of our study did not validate our hypothesis and we found low rates of both apoptosis and necrosis in the fallopian tubes after MTX therapy. After reviewing our methods, we found a few possible explanations for these results. First, there could have been inadequacy of the cleaved Caspase‐3 for the fallopian tube tissue or for the formaldehyde fixation. Second, the Methotrexate therapy may not have provoked a cellular reaction on the gestational tissue. Third, some studies have shown a time and dose‐dependent reaction to Methotrexate. As the MTX dose used is relatively low and exposure is short in ectopic pregnancies, it is possible that the reaction observed was therefore only partial. Fourth, both apoptosis and necrosis are gradual processes and so, if the procedures were preformed relatively soon after the MTX injection, it is possible that the full extent of the pathological reaction had yet to occur at the time of salpingectomy. Finally, the small sample size used in this study may have prevented us from reaching significant conclusions.

Ectopic pregnancies present one of the most frequent challenges to the practicing gynaecologist. Although Methotrexate is usually considered to be a valid treatment option for haemodynamically stable patients, the full pathophysiological mechanism and consequences following its administration remain unclear. It is in our collective interest to know and fully understand the full extent and possible sequalae of the treatments were administrating.

Although the results did not meet our exceptions, we believe our hypothesis may still have a foundation and that the importance of this subject is significant. This subject needs to be further evaluated. We will continue to pursue the matter and are searching for the right histopathological approach in order to prove our hypothesis. Our hypothesis that apoptosis rather than necrosis and hematoma formation is the dominant mechanism of self‐resolving ectopic pregnancies could be the explanation of fewer rupture cases after treatment, and could further promote our “watchful waiting” protocol in the treatment of ectopic pregnancies. We invite our colleagues to further investigate the subject and are willing to share our knowledge and experience in the field.

## AUTHOR CONTRIBUTIONS

YG, IL: Conception & design. YG, AZ, JB, AC: Acquisition & analysis of data. YG, AZ, IL: Manuscript/figures Drafting. JB: Others.

## FUNDING INFORMATION

No funding was received for this study.

## CONFLICT OF INTEREST

The authors have no conflicts of interest to report.
